# Tetranectin and Paraoxonase-1 as Markers of Heart Failure

**DOI:** 10.3390/medicina62020284

**Published:** 2026-01-31

**Authors:** Paula Alexandra Vulciu, Nicolae Catalin Valea, Dana Zdremtan, Chioreanu Alexandru, Norberth-Istvan Varga, Imola Donath-Miklos, Maria-Daniela Mot, Maria Puschita

**Affiliations:** 1 Department of Biochemistry, “Vasile Goldis” Western University, B-dul Revolutiei Nr. 96, 310025 Arad, Romania; vulciu.paula@uvvg.ro (P.A.V.); valeacatalin@yahoo.com (N.C.V.); zdremtan.dana@uvvg.ro (D.Z.); 2 Department of ENT, “Vasile Goldis” Western University, B-dul Revolutiei Nr. 96, 310025 Arad, Romania; chioreanu.alexandru@uvvg.ro; 3 Doctoral School, Department of General Medicine, “Victor Babes” University of Medicine and Pharmacy, Eftimie Murgu Square 2, 300041 Timisoara, Romania; norberth.varga@umft.ro; 4 Department of Physiology, “Vasile Goldis” Western University, B-dul Revolutiei Nr. 96, 310025 Arad, Romania; miklosimola@gmail.com; 5 Department of General Medicine, “Vasile Goldis” Western University, B-dul Revolutiei Nr. 96, 310025 Arad, Romania; 6 Department of Internal Medicine, “Vasile Goldis” Western University, B-dul Revolutiei Nr. 96, 310025 Arad, Romania; mpuschita.mp@gmail.com

**Keywords:** heart failure, tetranectin, paraoxonase-1, biomarkers, fibrosis, oxidative stress, extracellular matrix remodeling, risk stratification

## Abstract

*Background and Objectives*: This narrative review evaluates the potential of Tetranectin (TN) and Paraoxonase-1 (PON1) to bridge the gap between biological pathology and clinical risk stratification by mapping the “Fibrosis-Oxidative Axis”. *Materials and Methods*: A targeted literature search was conducted using Scopus, PubMed, and Google Scholar to identify studies examining the diagnostic and prognostic value of TN and PON1 in heart failure (HF). Evidence was synthesized qualitatively to analyze their roles in structural fibrosis and oxidative defense. *Results*: Tetranectin functions as a structural indicator, where its dynamics reflect fibroblast activation, extracellular matrix (ECM) deposition, and protein sequestration during tissue remodeling. On the other hand, PON1 serves as a functional metabolic barometer; its reduced activity correlates with systemic oxidative burden, loss of endothelial protection, and pro-inflammatory signaling. These markers capture a bidirectional pathology where oxidative injury drives fibrotic remodeling, which subsequently continue metabolic dysfunction. A dual-biomarker profile is proposed to stratify disease activity: early-stage metabolic stress (reduced PON1) precedes structural changes, while progressive HF involves active fibrosis (altered TN) alongside persistent oxidative injury. *Conclusions*: The combined assessment of TN and PON1 offers a complementary approach to HF profiling, potentially refining risk stratification beyond hemodynamic parameters. However, clinical implementation requires large-scale validation to address standardization issues and specificity limitations regarding multimorbidity.

## 1. Introduction

Heart failure (HF) is a major global health challenge, affecting estimates around 56 to 64 million people, and imposing substantial clinical and economic burdens, especially in the context of major demographic changes (more people reaching age 65 or more) [1,2]. This phenomenon causes prevalence to increase even though incidence has stabilized and is amplified by improved survival after myocardial infarction and better comorbidity management [3]. This leads to a clinical management paradox: while our understanding of HF pathophysiology, and associated conditions has evolved to recognize complex interconnected mechanisms—specifically chronic inflammation, mitochondrial dysfunction, and extracellular matrix (ECM) remodeling—our diagnostic tools have suffered minimal changes over the last years [4,5].

Current severity and risk stratification relies on either somewhat subjective, potentially inaccurate parameters such as patient history, observed/reported symptom severity and foundational biomarkers for detecting hemodynamic stress such as serum urea, CO_2_ and NT-proBNP [6,7]. While one can also account for ischemic changes in EKG, or ejection fraction (EF) categories, each one exhibits method-dependent limitations [8,9]. Focusing on current markers for HF risk/severity stratification, one can notice that such markers frequently lack accuracy in common clinical scenarios, including heart failure with preserved ejection fraction (HFpEF), obesity, renal dysfunction, and early-stage disease [10,11,12]. To be more precise, the current literature shows that natriuretic peptides reflect the consequence (hemodynamic load) rather than the upstream causes (oxidative imbalance, ECM turnover, and vascular dysfunction) [13,14]. This leaves clinicians with a diagnostic drawback regarding the biological drivers, potentially explaining the previously described paradox.

**Figure 1:**
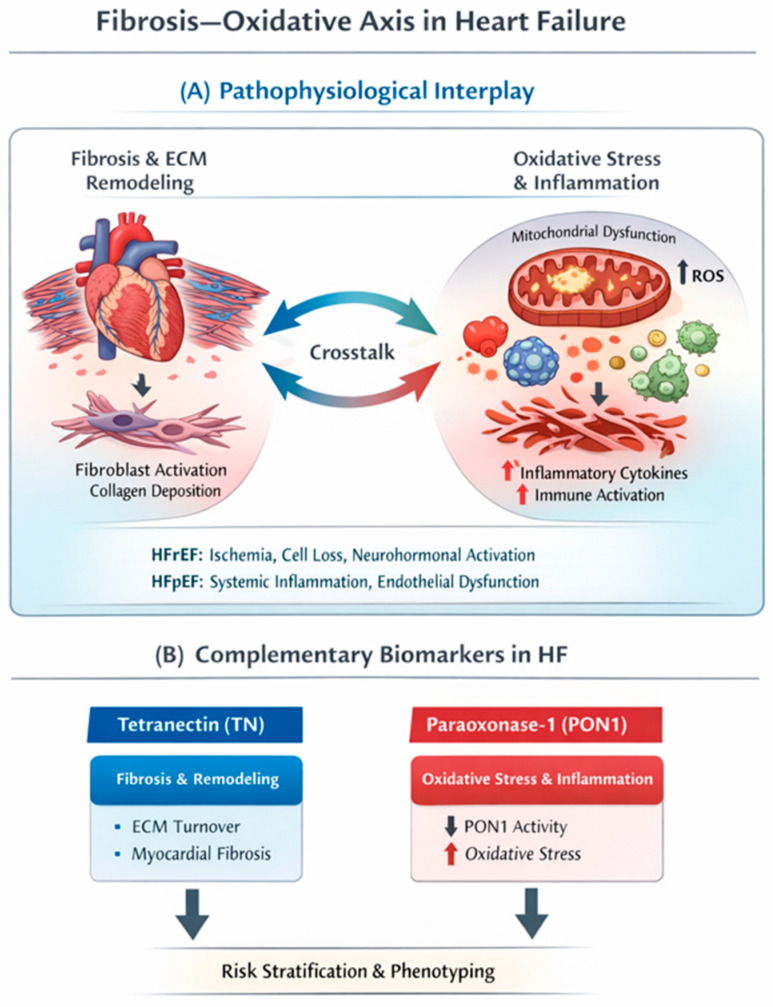
Fibrosis-Oxidative Axis in Heart Failure and Positioning or TN and PON-1. (**A**) Schematic representation of the bidirectional interaction between fibrotic extracellular matrix (ECM) remodeling and oxidative stress–inflammatory pathways in heart failure. Mitochondrial dysfunction, reactive oxygen species (ROS) generation, and inflammatory activation promote fibroblast activation and collagen deposition, while progressive fibrosis further exacerbates oxidative and inflammatory signaling. Dominant triggers may differ across heart failure phenotypes, with ischemia and cardiomyocyte loss prevailing in HFrEF and comorbidity-driven systemic inflammation and endothelial dysfunction in HFpEF. (**B**) Conceptual positioning of TN as a marker related to ECM turnover and myocardial remodeling, and PON1 as a marker reflecting oxidative stress and inflammatory milieu. Their combined assessment is proposed to support phenotyping and risk stratification in heart failure as an adjunct to established biomarkers.

Heart failure comprises heterogeneous phenotypes that share convergent biological drivers—oxidative injury, inflammation, and extracellular matrix remodeling—while differing in their dominant triggers across EF categories. In heart failure with reduced ejection fraction (HFrEF), cardiomyocyte loss (often ischemic) and neurohormonal activation promote mitochondrial dysfunction and reactive oxygen species (ROS) generation, with downstream redox-sensitive inflammatory signaling and progressive replacement/interstitial fibrosis contributing to systolic impairment. In HFpEF, comorbidity-driven systemic inflammation is thought to induce coronary microvascular endothelial activation and reduced nitric oxide bioavailability, thereby increasing cardiomyocyte stiffness and fostering diffuse interstitial fibrosis; oxidative stress acts as an amplifier of this inflammatory–fibrotic cascade [4,13]. The bidirectional interaction between fibrotic remodeling and oxidative–inflammatory pathways in heart failure, and the positioning of tetranectin and paraoxonase-1 within this framework, are schematically illustrated in Figure 1.

Against this pathophysiological background, recent advancements in proteomics highlight that oxidative damage and fibrotic activation form a shared biological axis driving both reduced and preserved ejection fraction phenotypes [15,16,17,18,19]. Consequently, the search for biomarkers has shifted toward candidates that can map these specific upstream domains [20]. Such biomarkers make the subject of this review in the form of Tetranectin (TN) and Paraoxonase-1 (PON1).

This study addresses a specific clinical question: Does the assessment of TN and Paraoxonase-1 offer superior diagnostic and prognostic resolution compared to, or alongside, traditional hemodynamic markers? A narrative review approach was selected because the available evidence on tetranectin and paraoxonase-1 spans heterogeneous clinical observational cohorts and mechanistic studies, with substantial variability in assays, populations, and endpoints that limits the feasibility of formal quantitative synthesis. Our primary aim is therefore conceptual integration and hypothesis generation (the proposed “Fibrosis–Oxidative Axis”) and clinical positioning of TN and PON1 as complementary markers.

## 2. Methods

For the database search, Scopus and PubMed were used as the primary platforms for structured, term-based queries, while Google Scholar was additionally used to capture broader results using natural-language search formulations. We used a combination of keywords related to the biomarkers and pathophysiological mechanisms: “TN”, “CLEC3B”, “Paraoxonase-1”, “PON1”, combined with “Heart Failure”, “Ejection Fraction”, “Fibrosis”, “Oxidative Stress”, and “Extracellular Matrix”.

The literature search covered publications from database inception through September 2025, and reference lists of key studies/reviews were hand-screened to identify additional relevant articles. Only studies with available English-language full texts were assessed. Human clinical studies (HF and related cardiovascular cohorts) were prioritized when discussing diagnostic/prognostic utility, whereas preclinical (animal) and in vitro studies were included when necessary to clarify TN/PON1 biology and pathways relevant to fibrosis and oxidative stress.

Titles and abstracts were screened by the authors for relevance to (i) clinical diagnostic/prognostic associations in HF or related cardiovascular cohorts and/or (ii) mechanistic links to oxidative stress and fibrotic remodeling, followed by full-text assessment of eligible papers. Because this is a narrative review, we did not aim to capture PRISMA-style record counts (retrieved/excluded) or perform a formal quantitative synthesis; instead, we prioritized representative and methodologically informative studies that directly address the research question.

**Figure 2:**
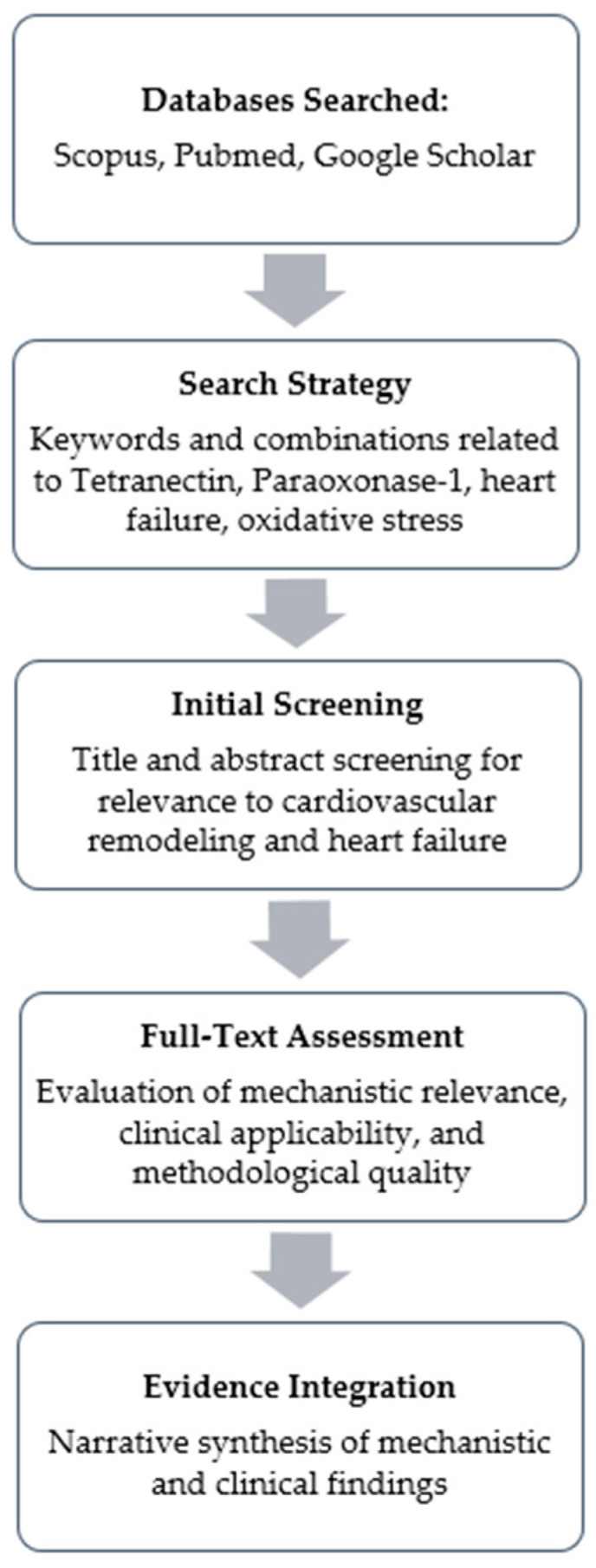
Workflow of Literature Search.

Figure 2 presents a flowchart of our search strategy.

## 3. Tetranectin

### 3.1. Discovery and Characteristics

Tetranectin was first identified and characterized in 1986 by Clemmensen and Petersen, following its isolation from human plasma during the purification of α_2_ plasmin inhibitor [21]. Initially distinct for its co-purification behavior, the protein was defined by its specific, high-affinity binding to the Kringle-4 domain of plasminogen. Structural analysis revealed a homotetrameric configuration consisting of four non-covalently linked polypeptide chains. This quaternary structure, combined with its binding capabilities, led to the designation “Tetranectin” (derived from the Greek “tetra”, meaning “four”, and “nectin”, meaning “to bind”).

Each subunit is approximately 17 kDa, yielding a total molecular mass of 68 kDa, mid-range size (similar to albumin and much smaller than large structural proteins) this suggests that circulating TN can move and turn over in the plasma compartment relatively efficiently, while still being large enough to participate in stable extracellular matrix and fibrinolytic interactions [22,23,24,25]. This balance may facilitate its release and detection as a biomarker of tissue remodeling and fibrosis in heart failure.

Biochemical profiling further demonstrated that TN binds to heparin and requires calcium ions (Ca^2+^) to maintain the conformational stability necessary for ligand interaction [21,26,27]. It is established that TN enhances the activation of Glu-plasminogen by tissue-type plasminogen activator (t-PA), suggesting a regulatory role in fibrinolysis and proteolytic activity [21,28].

Subsequent molecular studies have classified TN as a C-type lectin-like protein, encoded by the *CLEC3B* gene [29]. While predominantly synthesized in the liver and secreted into circulation, where it maintains stable plasma concentrations in healthy individuals, TN is also expressed in mesenchymal tissues, including bone, cartilage, or skeletal muscle, but also less expected places such as cerebrospinal fluid [23,25,30,31,32,33].

### 3.2. Molecular and Physiological Roles of Tetranectin

Building on fundamental biochemical properties, subsequent high-resolution structural analyses refined the understanding of TN’s architecture. It is worth mentioning that initially, it was identified as an oligomer, with later studies showing that the protein assembles via a trimeric α-helical coiled-coil core [34,35,36]. This specific configuration is functionally important, as it promotes cooperative ligand engagement and optimizes the spatial presentation of the C-type lectin domains for plasminogen activation [28,36]. Furthermore, the scope of TN’s interactome was found to extend beyond the plasminogen activation system. Expanded biochemical profiling revealed significant affinities for fibrin and various angiostatin fragments [37,38,39]. This evidence derives from experimental, tissue-expression, and in vitro studies.

Physiologically, the main contributor of circulating TN levels is the liver [25]. This was identified early through immunohistochemical investigations, with hepatocytes displaying a universal and distinct presence of the protein that suggested they are the primary source of circulating plasma levels [40]. Beyond hepatic production, TN synthesis is localized to specific tissues within the endocrine system, restricted to cells known for producing protein or glycoprotein hormones. Confirmed locations of synthesis include the chromophils of the pituitary gland, follicular and parafollicular cells of the thyroid, chief cells of the parathyroid, the Islets of Langerhans in the pancreas, and ganglion cells of the adrenal medulla. Conversely, TN is notably absent in steroid-producing tissues, such as the adrenal cortex and the Leydig cells of the testis [40]. In fact several studies have identified many tissues and cells with various degrees of TN expression [41,42,43,44,45]. However, of particular interest to our literature analysis is the demonstration that fibroblasts not only synthesize a TN-related protein (indistinguishable from plasma TN in molecular weight, 17 kDa) but actively secrete it into the surrounding environment [46]. Crucially, this protein does not merely diffuse; it is deposited and retained within the extracellular matrix structure. The mechanism of this retention appears to be mediated by TN’s affinity for sulphated polysaccharides, such as heparin, and proteoglycans, which are abundant in the connective tissue stroma [46].

While the fibroblast-matrix axis highlights TN’s role in structural scarring, pivotal work by Wewer et al. (1998) revealed that the protein is also fundamental to muscle regeneration and differentiation [44]. In healthy adult muscle, TN is virtually absent. However, during embryonic development and—most importantly—during the repair of damaged tissue, its expression is dramatically re-induced. By bridging the plasminogen activation system with ECM components, TN facilitates localized proteolysis essential for cell migration, tissue repair, and myogenesis; importantly this potential seems preserved for exogenous TN as well [44,47,48].

The biological function of TN in this context appears to be regulatory. In vitro studies using C2C12 myoblasts show that TN mRNA and protein are induced specifically during the differentiation phase, mediating interactions between the developing muscle cells and the extracellular matrix. More recent investigations have expanded this role to the myocardium, where *CLEC3B* (TN) overexpression has been shown to protect cardiomyocytes from hypoxia-induced apoptosis via the PI3K/Akt signaling pathway [49,50].

### 3.3. The Fibrosis-Oxidative Axis

The structural remodeling captured by TN does not occur in a vacuum; rather, it is inextricably linked to the metabolic environment of the myocardium, specifically the burden of oxidative stress. This relationship forms the “Fibrosis-Oxidative Axis,” a self-perpetuating cycle central to heart failure progression. Mechanistically, this interaction is bidirectional: reactive oxygen species (ROS) serve as potent signaling molecules that activate cardiac fibroblasts, driving the differentiation into myofibroblasts and followed by collagen deposition [18,51,52,53,54]. Consequently, capturing the full biological picture of heart failure requires assessing not just the downstream structural consequences (fibrosis) but also the upstream metabolic drivers [55]. This necessitates a biomarker capable of reflecting the systemic antioxidant capacity and oxidative burden—a role fulfilled by Paraoxonase-1.

### 3.4. Clinical Evidence of TN as a Biomarker in Cardiovascular Disease

Evidence from observational human studies suggests that circulating TN may have clinical utility as a remodeling-related biomarker across cardiovascular disease phenotypes. In stable coronary artery disease, a cross-sectional cohort study reported significantly lower serum TN in patients compared with healthy controls, with levels tracking angiographic disease burden and tissue staining showing higher TN expression in atherosclerotic arteries, supporting a relationship with vascular remodeling processes rather than a purely heart-specific signal [33]. In heart failure, an independent validation cohort demonstrated reduced circulating TN in HF compared with non-HF controls and showed strong discriminatory performance, while myocardial TN expression correlated with fibrotic signatures—together suggesting potential diagnostic value and mechanistic linkage to myocardial fibrosis/remodeling [49]. In cardio-oncology, a prospective cohort of women treated with anthracyclines found that among patients developing anthracycline-related cardiac dysfunction, lower TN was associated with an adverse clinical course during follow-up and provided prognostic information, including incremental value when combined with NT-proBNP [50]. Collectively, these studies support TN as a candidate marker for phenotyping and risk stratification in settings where extracellular matrix turnover and remodeling are clinically relevant, while also underscoring that its interpretation is phenotype- and context-dependent [33,49,50]. Emerging evidence also links tetranectin to metabolic disease biology, including type 2 diabetes, suggesting that diabetes/metabolic syndrome may influence baseline TN and should be considered when interpreting TN in HF populations [45].

## 4. Paraoxonase-1

### 4.1. Discovery and Characteristics

Paraoxonase-1 (PON1) was initially identified and named based on its toxicological capacity to hydrolyze organophosphate compounds, specifically the pesticide metabolite paraoxon [56]. However, this nomenclature obscures its primary physiological identity, which is also of better interest to the current review.

Biochemical characterization has since defined PON1 as a calcium-dependent hydrolase that circulates in plasma predominantly associated with high-density lipoproteins (HDL) [57]. This physical association is critical; the hydrophobic N-terminal signal peptide of PON1 anchors it to the HDL particle, a localized environment that is essential for maintaining the enzyme’s stability and active conformation [58]. PON1 possesses a broad catalytic profile that includes paraoxonase, arylesterase, and lactonase activities [59,60]. Current consensus posits that its lactonase activity represents its primary physiological function, mediating the hydrolysis of lipid lactones and oxidized phospholipids generated during metabolic stress [61].

### 4.2. Molecular and Physiological Roles of Paraoxonase-1

PON1 is a highly promiscuous enzyme capable of hydrolyzing diverse substrates including lactones, thiolactones, arylesters, organophosphates, cyclic carbonates, pharmaceutical agents like statins, and oxidized lipids [61,62]. Structure-reactivity studies suggest that PON1’s primary evolutionary function is lactonase activity, with paraoxonase activity being unexpected, since organophosphates are uncommon in nature [61,62]. The potency of this enzyme does not stop here, with proposed models linking lactonase activity to degradation of lipid peroxides: oxidized lipids containing hydroxyl groups at the 5′-position can be lactonized by PON1 to yield lysophosphatidylcholine and δ-valerolactone products [63]. These aspects are considered of interest in the evaluation of liver function.

To state it simply, PON1 is a marker of metabolic activity and oxidative stress. Hence the potential for cardiac function and cardiac insufficiency evaluation through the intrinsic potential of PON1 which appears to be degrading specific oxidized cholesteryl esters and oxidized phospholipids in lipoproteins and cell membranes, which accumulate during oxidative and metabolic stress conditions like inflammation, atherogenesis, and heart failure [63,64,65,66].

The relevance of PON1 to cardiovascular pathophysiology lies in its preservation of endothelial homeostasis [67,68]. Under normal conditions, PON1 acts as a guardian of nitric oxide (NO) bioavailability [69]. Mechanistically, oxidative modification of HDL—specifically by malondialdehyde (MDA)—enables it to activate the lectin-like oxidized LDL receptor-1 (LOX-1) and subsequently the PKCβII pathway, which inhibits endothelial NO synthase (eNOS) [70]. Functional PON1 breaks this chain by hydrolyzing the lipid peroxides before they can trigger this cascade [69,70].

Conversely, a loss of PON1 activity results in a pro-inflammatory environment. Reduced activity correlates with increased expression of adhesion molecules and inflammatory cytokines, including MCP-1, IL-6, and TNF-α [70,71]. Thus, PON1 sits at the nexus of oxidative stress regulation and inflammatory control, preventing the vascular injury that often precedes structural remodeling. In addition, PON1 directly suppresses macrophage pro-inflammatory responses, showing not only humoral potency but also direct cellular regulatory activity [72].

### 4.3. The Fibrosis-Oxidative Axis and Heart Failure

While TN maps the structural consequence of heart failure (fibrosis), PON1 maps the metabolic environment that facilitates it. Oxidative stress is a well-established driver of mitochondrial dysfunction and cardiomyocyte apoptosis, processes that trigger the replacement of functional myocardium with fibrotic tissue [13,54]. Recent combined analyses suggest that these markers provide complementary insights: low PON1 activity (oxidative susceptibility) correlates with elevated NT-proBNP and echocardiographic indices of remodeling, effectively identifying the metabolic conditions under which the structural fibrosis (indicated by TN) thrives [73,74,75]. Thus, while no direct protein-protein binding between PON1 and TN has been characterized, they are biologically linked through the shared pathology of ROS-induced tissue remodeling [19,55,75].

In the context of tissue hypoxia, PON1 activity serves as a barometer for the systemic oxidative burden [76]. On top of PON1 levels, clinical data demonstrate that serum arylesterase activity (a stable surrogate for PON1 function) is significantly reduced in patients with chronic systolic HF compared to healthy controls [77,78,79]. This reduction is not merely a marker of disease presence: lower arylesterase activity has been associated with worse heart failure–related outcomes and major adverse cardiac events (MACE), independent of traditional risk factors [79].

The suppression of PON1 in heart failure is likely mechanistic. Myeloperoxidase (MPO), an enzyme upregulated during cardiac stress, promotes oxidative modification of the HDL particle, disrupting the specific binding interface required for PON1 stability [80,81,82]. This creates a vicious cycle: oxidative stress displaces PON1, further reducing antioxidant capacity and accelerating myocardial injury [83]. Conversely, the mechanical stiffness of fibrotic ECM enhances mitochondrial dysfunction in cardiomyocytes, further amplifying ROS production. This creates a pathological feedback loop where oxidative damage initiates fibrosis, and established fibrosis sustains oxidative injury.

Implications regarding HF evaluation can be deduced from the presented biochemical implications and will be further discussed in the Section 5.

### 4.4. Clinical Context Beyond Heart Failure and Major Determinants of PON1 Activity

In human studies, reduced PON1 activity has been repeatedly associated with worse outcomes, including more severe coronary artery disease and higher event risk, consistent with its role as an HDL-linked antioxidant defense marker [79]. Importantly, PON1 status can also change with clinical trajectory: improvement in systemic oxidative balance after major interventions has been associated with increases in PON1 activity alongside favorable cardiac remodeling signals, supporting the concept that PON1 may serve as a dynamic readout of the oxidative environment in which remodeling occurs [74]. However, baseline PON1 activity varies substantially between individuals due to genetic polymorphisms and methodological differences between activity assays, which complicates direct comparison between studies unless the assay and phenotype are carefully specified [59,60,75].

Beyond cardiovascular disease, PON1 activity is broadly reduced across conditions characterized by oxidative stress and inflammation, including diabetes and insulin resistance states, chronic kidney disease, and sepsis. Because PON1 is synthesized in the liver and circulates bound to HDL, hepatic dysfunction and altered HDL composition may lower measured PON1 activity and should be considered alongside diabetes and renal dysfunction as major confounders in HF cohorts [63]. Noteworthy that these comorbidities frequently coexist with chronic heart failure and may confound biomarker interpretation [70,71,72,74,78]. For this reason, the clinical usefulness of PON1 in heart failure is strongest when interpreted as part of a broader risk profile (comorbidity burden, renal function, inflammatory state) and when paired with a structural remodeling marker such as TN rather than used as a stand-alone diagnostic discriminator [56,62,75,79].

## 5. Discussions

### 5.1. Diagnostic Utility

The conceptual framework of the “Fibrosis-Oxidative Axis,” as detailed in earlier sections, provides a compelling biological rationale for a dual-biomarker strategy in heart failure management. While traditional biomarkers such as NT-proBNP primarily reflect hemodynamic wall stress, they do not capture the specific upstream biological drivers of myocardial tissular status. By synthesizing the roles of TN and Paraoxonase-1 (PON1), a more comprehensive picture is revealed: TN predominantly reflects the structural endpoint of chronic injury—fibroblast activation, extracellular matrix (ECM) deposition, and tissue remodeling—whereas PON1 captures the profound metabolic intricacy, mainly systemic oxidative stress and its vascular consequences [84,85,86]. Monitoring these two distinct but interconnected domains may provide complementary, biologically informed information and could add incremental diagnostic and prognostic value beyond either marker alone; however, this remains a hypothesis that requires prospective validation.

From a clinical perspective, the advantages of this combined profile lie in its potential to stratify patients based on the active biological mechanism of their disease. Mechanistically, a patient presenting with reduced PON1 activity alongside altered TN levels could identify a phenotype of “active pathological remodelling,” where high oxidative stress is actively driving fibroblast differentiation and matrix deposition [53,54,75,77]. Theoretically, this biomarker profile might evolve dynamically across heart failure stages [87].

We propose the following trajectory as a working hypothesis for how a dual TN–PON1 profile might evolve across HF stages: in early disease, metabolic stress likely precedes overt structural changes, presenting as reduced PON1 activity with TN levels remaining within normal ranges. As the condition progresses to symptomatic heart failure, persistent oxidative injury (low PON1) may theoretically be accompanied by rising circulating TN, reflecting increased turnover and active secretion by fibroblasts into the extracellular environment. In advanced heart failure, a profile of high TN and severely suppressed PON1 may indicate established fibrosis exacerbated by unchecked oxidative injury, a state associated with poor outcomes. Consequently, this dual signature could refine risk stratification beyond current standards, potentially identifying patients who remain at high risk for adverse events despite normalized natriuretic peptides. Some human observational cohorts are consistent with elements of this proposed trajectory; however, EF-stratified, prospective studies are needed to validate temporal patterns and incremental value [77,87,88,89].

Furthermore, this approach opens the discussion for precision therapeutic monitoring: changes in TN could track the efficacy of antifibrotic interventions, while recovery of PON1 activity could serve as a specific metric for metabolic or antioxidant therapies, distinct from the hemodynamic responses monitored by standard agents [72,77]. Integrating a structural marker (TN) with a metabolic–oxidative marker (PON1) may therefore improve discrimination of high-risk patients, particularly in those whose NT-proBNP or troponin values are borderline or discordant with clinical status. Whether such a combined panel provides incremental predictive value over established biomarkers such as NT-proBNP, ST2, or galectin-3 should be formally tested in adequately powered, prospective cohorts [90,91,92,93,94].

### 5.2. Positioning Relative to Established Heart Failure Biomarkers

Clinically, TN and PON1 should be regarded as adjunctive biomarkers rather than replacements for established tests. Natriuretic peptides (BNP/NT-proBNP) remain central for diagnosis and congestion/hemodynamic stress assessment, while cardiac troponins primarily reflect myocardial injury; by contrast, TN and PON1 are proposed to capture upstream domains that are only indirectly represented by hemodynamic markers [7,13,14]. A practical interpretation is therefore phenotype enrichment and risk stratification: TN/PON1 may be most informative when natriuretic peptide interpretation is challenging (e.g., HFpEF, obesity, renal dysfunction) or when clinical status appears discordant with hemodynamic biomarkers [10,11,12]. At present, EF-specific cutoffs and differential performance across HFrEF vs. HFpEF are not well established because many available studies do not consistently stratify by EF phenotype; future prospective cohorts should explicitly test incremental value over natriuretic peptides and other established remodeling/fibrosis markers (e.g., ST2, galectin-3) within each EF category [15,16,17,18,19,90,91,92,93,94].

To contextualize TN and PON1 relative to established HF biomarkers, we provide a qualitative positioning overview summarizing the dominant biological domain captured, common clinical use, and major confounders (Table 1). Because available studies differ substantially in assays, populations, and endpoints, the table is intended for conceptual comparison rather than head-to-head performance ranking.

This overview supports viewing TN and PON1 primarily as adjunctive biomarkers that may add domain-specific information (remodeling and oxidative/inflammatory milieu) to established hemodynamic and injury markers, pending prospective validation.

### 5.3. Challenges and Future Directions

Despite the promising physiological plausibility, several limitations and challenges must be addressed before clinical implementation. First, while the individual prognostic values of TN and PON1 are supported by previously cited literature, direct prospective validation studies evaluating their combined diagnostic utility in large, diverse heart failure cohorts are missing.

To be fair, standardization presents another drawback; TN assays are not yet routinely available or harmonized across laboratories (monoclonal antibody-based immunoassay proposed as the most accurate), and PON1 assessment is complicated by the existence of multiple activity assays (lactonase vs. arylesterase) and genetic polymorphisms that influence baseline activity independent of disease state [59,60,75,95,96]. Specificity is also a concern, as neither marker is exclusive to cardiac pathology [25,40,71]. TN elevations or fluctuations may occur in other fibrotic conditions such as liver cirrhosis or cancer [97,98,99], while PON1 activity is non-specifically reduced in diverse states of oxidative stress, including diabetes, chronic kidney disease, and sepsis [100,101,102,103,104,105,106,107]. This is to be expected since processes such as fibrosis affect many organs and especially in the context of chronic HF, usually other organs functions are altered, a main example being that of the kidney [105,106].

Clinicians would therefore need to distinguish heart failure-specific changes from those driven by comorbidities. Finally, mechanistic gaps persist; while the relationship between PON1 and TN is inferred through parallel pathways of ROS-induced remodeling, no direct protein–protein interaction has been demonstrated, and the relative contribution of each marker to the overall prognostic picture requires further elucidation to determine if the cost-effectiveness of this multi-marker panel justifies its use over established tools like NT-proBNP, galectin-3 or ST2 [108,109,110].

Future studies should prioritize prospective, EF-stratified cohorts with prespecified endpoints to test whether TN and PON1 provide incremental value over natriuretic peptides and established remodeling biomarkers, ideally using standardized assays and serial sampling to assess temporal dynamics. Analyses should prespecify adjustment/stratification by major comorbidities (liver disease, diabetes/metabolic status, renal dysfunction) and evaluate whether a combined TN–PON1 profile improves phenotyping and risk prediction compared with single-marker models.

In summary, the combined assessment of TN and PON1 offers an attractive conceptual hypothesis for capturing both structural and metabolic dimensions of the Fibrosis–Oxidative Axis in heart failure, with potential advantages for diagnosis, staging, risk stratification, and even therapeutic monitoring. At the same time, the current evidence base is largely indirect and methodologically heterogeneous, and substantial work is required to validate this dual-biomarker approach before it can be recommended for routine clinical use.

## 6. Conclusions

Tetranectin and Paraoxonase-1 may provide complementary insights into heart failure pathophysiology. TN is proposed as a remodeling-related marker associated with fibroblast activation, extracellular matrix (ECM) deposition, and protein sequestration during tissue remodeling, whereas PON1 activity reflects aspects of the systemic oxidative–inflammatory milieu and loss of HDL-linked endothelial protective mechanisms. Together, these biomarkers can be positioned within a conceptual “Fibrosis–Oxidative Axis”, in which oxidative injury and inflammation promote fibrotic remodeling, which may in turn exacerbate metabolic and endothelial dysfunction.

The potential clinical value of a dual TN–PON1 profile lies in phenotyping and risk stratification by biological disease activity rather than symptom severity alone; however, this remains a hypothesis supported primarily by associative clinical evidence and requires prospective validation. Important limitations must be addressed before translation: neither biomarker is cardiac-specific, TN may vary with liver disease and malignancy, and PON1 activity is influenced by diabetes/metabolic status, renal dysfunction, hepatic function, and assay-related variability, necessitating careful interpretation in multimorbid populations.

Future research should prioritize assay harmonization and adequately powered, EF-stratified prospective cohorts to determine whether this multi-marker approach provides incremental prognostic and phenotyping value over established biomarkers of cardiac function.

## Figures and Tables

**Table 1:** Qualitative Positioning of TN and PON-1 Relative to Established HF Biomarkers. HF = heart failure; ECM = extracellular matrix; BNP = B-type natriuretic peptide; NT-proBNP = N-terminal proBNP; sST2 = soluble suppression of tumorigenicity 2; CKD = chronic kidney disease.

Biomarker	Dominant Domain Captured	Typical Current Clinical Role in HF	Key Confounders/Limitations	Where TN/PON1 May Add Complementary Value
BNP/NT-proBNP	Hemodynamic wall stress/congestion	Diagnosis support, prognosis, therapy monitoring	Affected by age, obesity, renal dysfunction, atrial fibrillation; reflects consequence rather than upstream drivers	TN/PON1 aim to add upstream biology (remodeling + oxidative/inflammatory milieu) when NP interpretation is challenging
High-sensitivity troponin	Myocardial injury	Risk stratification; identifies ongoing injury	Elevated in CKD, sepsis, tachyarrhythmias; not specific to remodeling mechanism	TN (remodeling) + PON1 (oxidative milieu) may contextualize injury-driven progression
sST2	Stress/inflammation; remodeling signaling	Prognosis/risk stratification	Influenced by systemic inflammation; assay/platform differences	TN may provide ECM/remodeling-related signal; PON1 adds oxidative/HDL-linked antioxidant domain
Galectin-3	Fibrosis/macrophage activation	Prognosis; fibrosis-related risk enrichment	Influenced by renal dysfunction and systemic fibrotic/inflammatory states	TN provides an alternative remodeling-related marker; pairing with PON1 adds oxidative–inflammatory component
Tetranectin (TN)	ECM turnover/remodeling (fibrosis-related)	Emerging: phenotyping/risk enrichment in remodeling-dominant HF	Not cardiac-specific; influenced by liver function and malignancy; assay availability/harmonization	Structural/remodeling axis component—potentially complements hemodynamic and injury markers
Paraoxonase-1 (PON1) activity	Oxidative stress/inflammatory milieu (HDL-linked antioxidant function)	Emerging: risk enrichment, oxidative/inflammatory profiling	Strongly affected by diabetes/metabolic status, renal dysfunction, liver function, genotype, and assay substrate choice	Functional/oxidative axis component—potentially complements TN (structure) and natriuretic peptides (hemodynamics)

## Data Availability

No new data were created or analyzed in this study.

## Abbreviations

The following abbreviations are used in this manuscript:

HF	Heart failure
HFpEF	Heart failure with preserved ejection fraction
EF	Ejection fraction
ECM	Extracellular matrix
CO_2_	Carbon dioxide
NT-proBNP	N-terminal pro–B-type natriuretic peptide
EKG	Electrocardiogram
TN	Tetranectin
PON1	Paraoxonase-1
CLEC3B	C-type lectin domain family 3 member B (gene encoding TN)
HDL	High-density lipoprotein
LDL	Low-density lipoprotein
NO	Nitric oxide
ROS	Reactive oxygen species
MDA	Malondialdehyde
LOX-1	Lectin-like oxidized low-density lipoprotein receptor-1
PKCβII	Protein kinase C beta II isoform
eNOS	Endothelial nitric oxide synthase
MCP-1	Monocyte chemoattractant protein-1
IL-6	Interleukin-6
TNF-α	Tumor necrosis factor alpha
MPO	Myeloperoxidase
MACE	Major adverse cardiac events
ST2	Suppression of tumorigenicity 2 (IL-33 receptor family member)
PI3K	Phosphoinositide 3-kinase
Akt	Protein kinase B
t-PA	Tissue-type plasminogen activator
C2C12	Mouse myoblast cell line (C2C12)
